# PulseNet: the molecular subtyping network for foodborne bacterial disease surveillance, United States.

**DOI:** 10.3201/eid0703.010303

**Published:** 2001

**Authors:** B Swaminathan, T J Barrett, S B Hunter, R V Tauxe, CDC PulseNet Task Force

**Affiliations:** Centers for Disease Control and Prevention, Atlanta, Georgia 30333, USA. bas5@cdc.gov

## Abstract

PulseNet, the national molecular subtyping network for foodborne disease surveillance, was established by the Centers for Disease Control and Prevention and several state health department laboratories to facilitate subtyping bacterial foodborne pathogens for epidemiologic purposes. PulseNet, which began in 1996 with 10 laboratories typing a single pathogen (Escherichia coli O157:H7), now includes 46 state and 2 local public health laboratories and the food safety laboratories of the U.S. Food and Drug Administration and the U.S. Department of Agriculture. Four foodborne pathogens (E. coli O157:H7; nontyphoidal Salmonella serotypes, Listeria monocytogenes and Shigella) are being subtyped, and other bacterial, viral, and parasitic organisms will be added soon.


### PulseNet: The Molecular Subtyping Network for Foodborne Bacterial Disease Surveillance, United States

**Bala Swaminathan, Timothy J. Barrett, Susan B. Hunter, Robert V. Tauxe, and the CDC PulseNet Task Force[1]**
Centers for Disease Control and Prevention, Atlanta, Georgia USA

PulseNet, the national molecular subtyping network for foodborne disease surveillance, was established by the Centers for Disease Control and Prevention and several state health department laboratories to facilitate subtyping bacterial foodborne pathogens for epidemiologic purposes. PulseNet, which began in 1996 with 10 laboratories typing a single pathogen (*Escherichia coli* O157:H7), now includes 46 state and 2 local public health laboratories and the food safety laboratories of the U.S. Food and Drug Administration and the U.S. Department of Agriculture. Four foodborne pathogens (*E. coli* O157:H7; nontyphoidal *Salmonella* serotypes, *Listeria monocytogenes* and *Shigella*) are being subtyped, and other bacterial, viral, and parasitic organisms will be added soon.

Molecular subtyping of bacterial isolates by characterization of proteins or nucleic acids has been successfully applied to aid epidemiologic investigations of foodborne disease outbreaks since the initial use of plasmid fingerprinting nearly 20 years ago (1,2). Since that time, several methods for identifying restriction fragment length polymorphisms on chromosomal DNA have been developed, and molecular subtyping has become an essential component of epidemiologic investigations of infectious diseases (3-10).

This widespread use of molecular typing has resulted in a plethora of techniques and protocols for subtyping even the same species of bacteria (11). Because each laboratory uses its own protocols for molecular typing and designations of patterns, the results cannot be compared with those of another laboratory, even if both laboratories have used essentially the same methods. This lack of comparability has greatly diminished the power of molecular subtyping methods.

In 1993, during the investigation of an *Escherichia coli* O157:H7 outbreak caused by contaminated hamburgers served in a fast-food restaurant chain in the western United States, Barrett et al. in our laboratory applied pulsed-field gel electrophoresis (PFGE) to characterize clinical and food isolates of *E. coli* O157:H7 and demonstrated its utility in outbreak investigations (12). Subsequently, our laboratory received numerous requests from state health departments for subtyping *E. coli* O157:H7. The demand soon overwhelmed our testing capacity, and delays in subtyping isolates meant that results were mostly useful only for laboratory confirmation of conclusions from epidemiologic investigations. We reasoned that decentralization of subtyping activities and transfer of standardized molecular subtyping methodology to public health laboratories should enable more timely subtyping of clinical and food isolates. One result would be information useful to epidemiologists while they were investigating outbreaks. In addition, routine subtyping of isolates of foodborne pathogenic bacteria received by public health laboratories should lead to identification of outbreaks not readily recognizable by other means. Use of standardized subtyping methods would allow isolates to be compared from different parts of the country, enabling recognition of nationwide outbreaks attributable to a common source of infection, particularly those in which cases are geographically separated.

In 1995, the Centers for Disease Control and Prevention (CDC), with the assistance of the Association of Public Health Laboratories (APHL), selected the state public health laboratories in Massachusetts, Minnesota, Washington, and Texas as area laboratories for a national molecular subtyping network for foodborne bacterial disease surveillance. This network later became known as PulseNet (13). Standardized PFGE typing and pattern analysis technology would be transferred to the area laboratories, which would assume responsibility for subtyping foodborne pathogenic bacteria from their states and providing subtyping service to neighboring states that requested assistance. At about the same time, CDC and five state health departments, as part of a response to emerging infectious disease threats (14), implemented an active foodborne disease surveillance program called FoodNet (15). The objectives of FoodNet were to accurately estimate the burden of foodborne disease in the United States, investigate the sources of infection in outbreaks and sporadic cases, and build public health infrastructure for dealing with emerging foodborne disease issues. In 1996, FoodNet included Minnesota, Oregon,

Address for correspondence: Bala Swaminathan, Foodborne and Diarrheal Diseases Branch, Centers for Disease Control and Prevention, 1600 Clifton Road, Mailstop C03, Atlanta, GA 30333, USA; fax: 404-639-3333; e-mail: bas5@cdc.gov

[1]The CDC PulseNet Task Force includes John Allan, Michelle Bird, Daniel Cameron, Wallis DeWitt, Thomas Donkar, Collette Fitzgerald, Judy Gaither, Lewis Graves, Peggy Hayes, Jared Kerr, Kristy Kubota, Mary Ann Lambert-Fair, Susan Maslanka, Loretta McCroskey, Jeremy Miller, Heather Noll, Efrain Ribot, Nicole Tucker, Susan Van Duyne, and the authors.

Connecticut, and Georgia and selected counties in California. Participants in FoodNet recognized the advantages offered by PulseNet, and the public health laboratories in Oregon and Georgia began participating in PulseNet. The first 5-day workshop on standardized methods for PFGE for foodborne pathogenic bacteria was held in January 1996. By early 2000, PulseNet included 46 state public health laboratories, the public health laboratories in New York City and Los Angeles County, California, the U.S. Department of Agriculture's Food Safety and Inspection Service Laboratory (USDA-FSIS) and the U.S. Food and Drug Administration laboratories in the Center for Food Safety and Applied Nutrition (FDA-CFSAN) and Center for Veterinary Medicine (Figure 1). In addition, six provincial Canadian laboratories joined PulseNet in 1999-2000; their participation is coordinated through the National Laboratory for Enteric Pathogens, Canadian Science Centre for Human and Animal Health Winnipeg, Manitoba.

As PulseNet's capacity expands, the need for epidemiologic assessment of new information expands in parallel because timely evaluation of clusters identified by the network is critical and warranted. PulseNet's laboratory evaluation of isolates from clusters or outbreaks identified through routine epidemiologic surveillance has already demonstrated its value in early recognition of outbreaks and rapid identification of their sources. A welcome consequence is engendering close collaboration between epidemiologists and microbiologists throughout the public health system.

### Standard Protocols

During 1996 and early 1997, we evaluated the standard protocol for *E. coli* O157:H7 at participating PulseNet laboratories. The original protocol, similar to the one used by Barrett et al. (12), required 3 to 4 days of testing; it involved an overnight incubation for cell lysis and another for restriction of chromosomal DNA. A set of 64 *E. coli* O157:H7 strains was compiled to evaluate the reproducibility of DNA fingerprint patterns in different laboratories. This set was sent to participating laboratories, which were asked to type strains by using the standardized protocol and return the raw electronic images of PFGE patterns to a common CDC database for study. Data analysis showed that when the standardized protocol is strictly followed by participating laboratories, results are highly reproducible and DNA patterns generated at different laboratories can be compared (Table 1). Also included in this set were duplicates of nine isolates to assess intralaboratory reproducibility of PFGE patterns; the testing laboratories were unaware of the duplicate strains until results were analyzed and reported. For six of nine sets, all laboratories generated patterns that were exact matches within each set. For each of the three remaining sets of duplicates, one of seven laboratories did not generate an exact match but matched the duplicates at 95%-97% similarity.

### Standardized Equipment for Participating Laboratories

PulseNet laboratories use CHEF-DRII, CHEF-DRIII, or CHEF-Mapper (Bio-Rad Laboratories, Hercules, CA) for PFGE of restricted bacterial DNA. Although all three instruments can run PulseNet protocols, CHEF-Mapper allows greater flexibility in development of electrophoretic separation conditions and nonlinear ramping. After electrophoresis, the gels are stained with ethidium bromide, and PFGE patterns are digitized in a TIFF format (uncompressed .tif file) by using a Gel-Doc 1000 (replaced by Gel-Doc 2000; Bio-Rad Laboratories) or other image acquisition equipment capable of 768 x 640 pixels or higher resolution. Molecular Analyst Fingerprinting Plus with Data Sharing Tools (MAFP-DST; Bio-Rad Laboratories; sold as GelCompar in Europe) is the software program used by PulseNet laboratories for analysis of PFGE patterns. MAFP-DST is being replaced with BioNumerics software (Applied

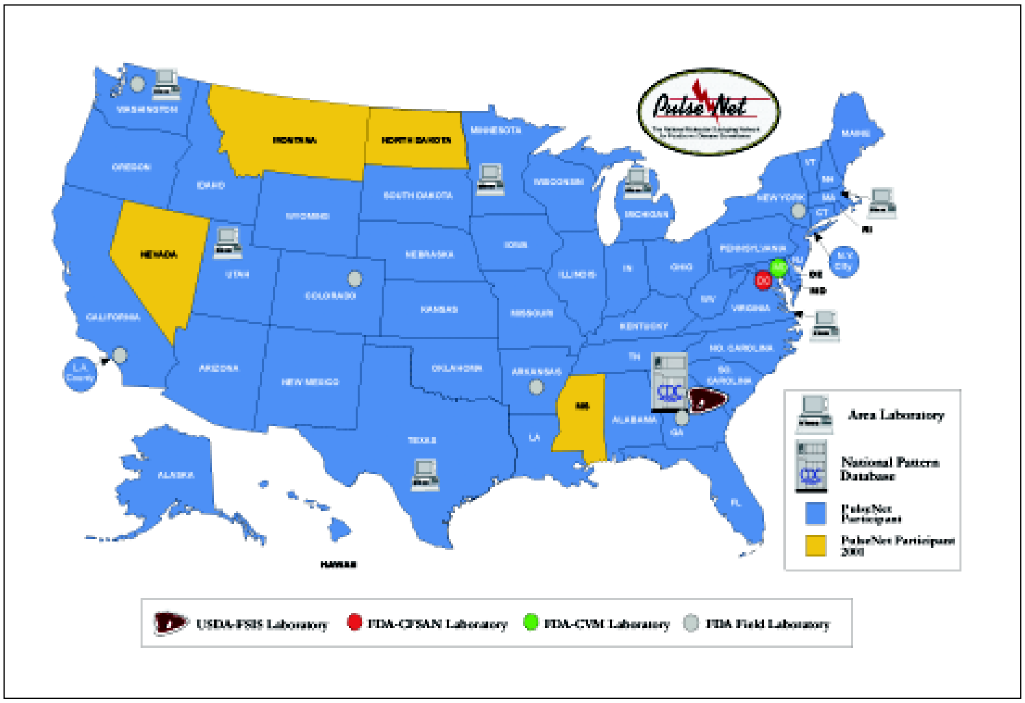

Figure 1. Locations of PulseNet laboratories in the United States. PulseNet participant states are currently participating. States labeled PulseNet participants 2001 are expected to complete the requirements for entry by December 2001. The area laboratories provide surge capacity and technical support to neighboring states. FDA-CFSAN: U.S. Food and Drug Administration, Center for Food Safety and Applied Nutrition Laboratory; FDA-CVM: U.S. Food and Drug Administration, Center for Veterinary Medicine Laboratory; USDA-FSIS: U.S. Department of Agriculture, Food Safety and Inspection Service Laboratory.

Table 1. Interlaboratory reproducibility of pulsed-field gel electrophoresis patterns of 64 *Escherichia coli* isolates by eight laboratories following the PulseNet standardized protocol

| | Laboratory (%) | | | | | | | |
|---|---|---|---|---|---|---|---|---|
| Result | 1 | 2 | 3 | 4 | 5 | 6 | 7 | 8 |
| Matched expected pattern | 64/64[a] (100) | 63/63 (100) | 59/63 (93.7) | 62/64 (96.9) | 62/64 (96.9) | 62/64 (96.9) | 61/64 (95.3) | 61/64 (95.3) |
| $\leq$ 1-band difference from expected pattern | 64/64 (100) | 63/63 (100) | 62/63 (98.4) | 64/64 (100) | 63/64 (98.4) | 64/64 (100) | 64/64 (100) | 64/64 (100) |

[a]Values represent no. of patterns fitting in the specified category/no. of isolates tested.

Maths, Kortrijk, Belgium); the change-over will be completed in 2001. Each PulseNet laboratory has all the above equipment and has the capability to normalize the patterns, compare them with other patterns, and maintain local databases of PFGE patterns for each bacterial pathogen of interest.

### National Database of PFGE Patterns and Associated Epidemiologic Information

A national database of PFGE patterns is being assembled for foodborne bacterial pathogens. These databases reside on a PulseNet server at CDC. For each bacterial pathogen, the normalized PFGE pattern is associated with a pattern database and a database of epidemiologic and clinical information for isolates. One isolate may be associated with more than one PFGE pattern in the database because PulseNet protocols may call for the use of more than one restriction enzyme to achieve appropriate discrimination between epidemiologically unrelated isolates. The *E. coli* O157:H7 database is functional; databases for nontyphoidal *Salmonella* serotypes and *Listeria monocytogenes* are under construction.

Seven PulseNet laboratories (four state public health laboratories, FDA-CFSAN, USDA-FSIS, and CDC) have direct access to the PulseNet database server through the Internet, enabling them to submit normalized PFGE patterns and associated epidemiologic information. (The DST version of the Molecular Analyst software creates a special “bundle” file for comparison with the national database.) Laboratories query the national database for identical matches or closely related patterns (>95% related under specified conditions). If identical or close matches to the submitted patterns are found, the submitting laboratory can access epidemiologic information associated with those patterns from the text database. When a PulseNet participating laboratory logs on to the PulseNet server, it will display a “recent match” message if two or more laboratories submit identical or closely related patterns within a specified time. This alert provides an early warning to PulseNet laboratories about possible multisite foodborne disease outbreaks.

PulseNet laboratories that do not yet have direct online access to the PulseNet server may still electronically submit raw TIFF images and normalized PFGE patterns (bundle files) to the PulseNet database administration team by e-mail or through file transfer protocols (ftp). The team compares the submitted patterns with those in the national database and e-mails the results to the submitting laboratory as quickly as possible. We expect that direct access to the PulseNet server will be available to all participating laboratories that have satisfactorily completed certification requirements by June 30, 2001.

### Developing Standardized Protocols

Standardized protocols for foodborne bacterial pathogens were developed in priority order based on the ability of PFGE to discriminate among strains of the organism and the epidemiologic utility of the resulting data. Standardized PFGE protocols have been developed for *E. coli* O157:H7, *Salmonella enterica* serotype Typhimurium, *L. monocytogenes*, and *Shigella* species. The *S.* Typhimurium protocol is applicable to most other nontyphoidal *Salmonella* serotypes, including *S.* Enteritidis. However, neither PFGE nor other molecular subtyping methods provide acceptable discrimination among strains of this highly clonal serotype. Standard PFGE protocols for *Campylobacter jejuni, C. coli*, and *Clostridium perfringens* (7) are being developed and validated. Although *C. jejuni* and *C. coli* infections are common, developing a standardized PFGE protocol for these organisms was not a high priority because they infrequently cause outbreaks. On the other hand, although outbreaks of *C. perfringens* infections are seldom widespread, state and local public health laboratories requested a standardized subtyping protocol to assist with local outbreak investigations. All PulseNet protocols are 1-day procedures based on the PFGE protocol developed by the Washington State Public Health Laboratory in response to the need for more rapid techniques (16). All new protocols and modifications of existing protocols are evaluated initially at the developing laboratory, followed by a second evaluation at CDC, alpha-testing at one or two PulseNet laboratories, and beta-testing at several PulseNet laboratories before they are adopted as official PulseNet protocols. Evaluation criteria include reproducibility of patterns, appropriateness of the strain used as the reference standard, and robustness of the procedure. Once a protocol is officially adopted, no changes can be made except by a petition to CDC’s PulseNet Task Force, discussion of the proposed changes, and adoption of the proposal by PulseNet laboratories. The PulseNet Task Force at CDC is composed of personnel who carry out PulseNet-related activities. The Task Force members develop and evaluate protocols, provide technical support for participating laboratories, organize and conduct training workshops, administer the certification program and proficiency testing program, and maintain the national databases of PFGE patterns for the bacteria under surveillance in PulseNet.

### Quality Control and Assurance Program

A quality assurance program has been instituted for PulseNet to ensure the integrity of results obtained with the standardized PFGE techniques. This program requires strict adherence to the standardized PFGE protocols (17). In addition, the quality assurance program consists of standards for training, analytical procedures, documentation, and equipment; standard operating procedures; an initial certification set of isolates for each organism; and an ongoing proficiency testing program. The standards detail the minimum requirements a laboratory must meet for training personnel, analytical procedures, documentation, equipment calibration and maintenance, proficiency testing, and review of results. The laboratory standard operating procedures describe procedures for record keeping, equipment maintenance, gel image acquisition, data analysis, and administrative policies. The certification sets consist of isolates with known patterns, which are sent to each laboratory. Laboratories type the isolates by the standardized protocol and send the gel images to the PulseNet National Database Administration Team for review. This team, part of the PulseNet Task Force, is responsible for maintaining and updating the PFGE pattern databases for foodborne disease-causing bacteria. Team members review new patterns submitted to the databases and verify matches. In addition, this team evaluates the certification data submitted by PulseNet laboratories. Laboratories with DST version of the MAFP software also analyze their gel images and send the results (bundle files) to the PulseNet National Database Administration Team for review. This team checks gel images and bundle files against the master certification set to ensure that the laboratory has obtained the correct patterns. Successful completion of the certification set allows PulseNet-affiliated laboratories to compare results with the National Database. As part of the proficiency testing program, laboratories will be sent a combination of isolates and TIFF files on a semiannual basis both to test the laboratory's ability to perform the standardized protocol correctly and to ensure that data analysis is consistent from laboratory to laboratory.

A quality assurance and control manual, being developed, will describe standardized training, laboratory and administrative procedures, and policies. A proficiency testing manual, also in preparation, is designed to maintain the reproducibility of patterns and consistency in analysis of patterns that make PulseNet a valuable ally for epidemiologists.

Laboratories joining PulseNet are sent the standardized PFGE protocols and certification sets appropriate to the organism(s) being tested. Appropriate training is scheduled and follow-up is provided by means of the certification sets and the regularly scheduled proficiency testing program. An annual meeting enables microbiologists from participating PulseNet laboratories to discuss new protocols and software upgrades and exchange information on problems and solutions.

### Standardized Nomenclature for PFGE Patterns

A major problem in comparing and interpreting molecular subtyping information from different laboratories has been the lack of a universal naming system for PFGE patterns. In response, we have developed a standardized nomenclature system for designating PFGE patterns in PulseNet. Each unique pattern in the database is represented by a 10-character code as follows:

XXXYYY.0000

The first three characters in the code represent the bacterial pathogen, the next three characters denote the enzyme used for DNA restriction, and the last four characters represent the pattern designation. For example, in the pattern designation EXHA26.0026, EXH represents *E. coli* O157:H7, A26 represents restriction endonuclease *Avr*II, and 0026 is the pattern number. Because the pattern numbers are assigned sequentially to unique patterns, no evolutionary, phylogenetic, or clonal relationships should be implied from the order of pattern numbers.

A priority order has been developed for inclusion of foodborne bacterial pathogens in PulseNet (Table 2). The prioritization takes into account the availability of an acceptable molecular subtyping method for a pathogen, severity of disease caused by that pathogen, propensity for the pathogen to cause outbreaks, and the potential for recognizing outbreaks and taking preventive action by routine subtyping.

Table 2. Priority order for inclusion of foodborne bacterial pathogens in PulseNet

| Pathogen | Expected year of inclusion |
|---|---|
| *Escherichia coli* O157:H7 | 1997 |
| Nontyphoidal *Salmonella* serotypes | 1998 |
| *Listeria monocytogenes* | 1999 |
| *Shigella sonnei* | 1999 |
| *Clostridium perfringens* | 2001 |
| *Campylobacter jejuni* | 2001 |
| *Vibrio parahaemolyticus* | 2001 |
| *V. cholerae* | 2001 |
| *Clostridium botulinum* | 2002 |
| Other pathogenic *E. coli* | 2002 |
| *Yersinia enterocolitica* | 2003 |

### Role of PulseNet in Outbreak Investigations

PulseNet plays several roles in detecting, investigating, and controlling outbreaks. Identification by PulseNet of an increase in a specific subtype of a pathogen may be an early indication of an outbreak. PFGE patterns submitted to the national database by participating laboratories may link apparently unrelated cases that are geographically dispersed. Once a cluster is detected through PulseNet, an epidemiologic investigation is initiated to determine if there is a common source. This epidemiologic investigation may be guided by the PFGE subtypes identified through PulseNet. PulseNet can identify outlier cases in other areas and define the geographic scope of the outbreak. If a common food source is identified and the pathogen is isolated from that food, subtyping helps confirm it as the outbreak strain. Finally, once control measures are instituted, PulseNet can help confirm that the outbreak is over by showing a substantial decrease in circulation of the outbreak strain in the affected communities. The following examples illustrate these PulseNet functions.

In 1996, epidemiologists at the Washington State and Seattle-King County health departments traced an outbreak of *E. coli* O157:H7 infections in four states and one Canadian province to commercial unpasteurized apple juice. Of 70 persons identified as part of this outbreak, 25 required hospitalization, 14 had hemolytic uremic syndrome, and one died. DNA fingerprinting by PFGE at the Washington State Public Health Laboratory, a PulseNet area laboratory, showed that isolates from patients and the apple juice were the same strain. Prompt recognition of the apple juice as the source of this outbreak resulted in rapid recall of the widely distributed product (18).

In 1997, the Colorado State Public Health Laboratory, which had just initiated PFGE typing of *E. coli* O157:H7, identified a cluster of 14 ill persons whose *E. coli* O157:H7 isolates had matching PFGE patterns. About the same time, the USDA laboratory isolated an *E. coli* O157:H7 strain from a ground beef patty from the same package as a patty eaten by an ill person. DNA fingerprinting by PFGE on the human isolate from Colorado and the food isolate from USDA-FSIS were generated by the PulseNet standardized protocol. The PFGE patterns were transmitted electronically to CDC via the Internet, where they were found to be indistinguishable. This outbreak pattern was then transmitted to PulseNet sites and compared with patterns from >300 other recent *E. coli* O157:H7 isolates. No matching patterns were found other than one case in Kentucky, providing strong evidence that the outbreak was not nationwide.

In May 1998, PulseNet facilitated the investigation of two clusters of *E. coli* O157:H7 infections in the northeastern United States. Timely fingerprinting of *E. coli* O157:H7 isolates by the Massachusetts Area Laboratory for PulseNet and other PulseNet laboratories in that region revealed two simultaneous clusters of *E. coli* O157:H7 infections (32 isolates in four of five states with one PFGE pattern and 25 isolates in all of five states with a second PFGE pattern), one of which could be traced to two supermarkets that received ground beef from the same distributor. Without assistance from PulseNet, epidemiologists would have found it difficult to identify cases associated with each cluster.

Also in May 1998, the state public health departments in both Illinois and Pennsylvania informed CDC about increases in *Salmonella* Agona infections. Serotype-specific surveillance data from other states quickly confirmed that 10 states had increases in *S.* Agona infections. A national outbreak of *S.* Agona was occurring, with no obvious source. Subsequently, the outbreak was traced to contaminated ready-to-eat toasted oats cereal product from a food-processing facility in Minnesota (19). PulseNet laboratories helped in this investigation by distinguishing cases that were associated with the outbreak from those that were not. In addition, timely PFGE typing of *S.* Agona by PulseNet laboratories helped identify outbreak-associated cases in states where the contaminated product was not initially thought to have been distributed. PFGE subtyping of *S.* Agona isolates was important in confirming the successful control of the outbreak. Not only did the number of reported isolates return to baseline, but also the outbreak strain disappeared. By the time this investigation was completed, PulseNet laboratories had typed >1,000 isolates of *S.* Agona. Four hundred nine cases (one fatal) in 23 states were linked to this outbreak (CDC, unpub. data).

From October 20 to November 9, 1998, health officials in Connecticut, New York, Ohio, and Tennessee reported increases in *Listeria* infections in their states (20). PFGE typing by PulseNet laboratories showed that several case isolates from different states had indistinguishable DNA fingerprints. On further investigation, 101 *Listeria* infections (including 15 perinatal infections) with bacteria having the same or highly similar DNA fingerprints were identified in 22 states. Fifteen deaths and six miscarriages or stillbirths were reported among patients who were infected with the outbreak strain. This outbreak was traced to contaminated hot dogs and sandwich meat produced at a single large meat-processing plant in Michigan (21). After the company voluntarily recalled the implicated lots of product and suspended production, the outbreak rapidly ended.

### Surveillance for Foodborne Outbreaks

Twenty years ago, most foodborne outbreaks were local problems that typically resulted from improper food-handling practices. Outbreaks were often associated with individual restaurants or social events and often came to the attention of local public health officials through calls from affected persons. These persons, who may have known others who had become ill after eating a shared meal or visiting the same restaurant, provided health officials with much of the information needed to begin an investigation.

Today, foodborne disease outbreaks commonly involve widely distributed food products that are contaminated before distribution, resulting in cases that are spread over several states or countries. It is less common for ill persons to know others who were ill or to be able to identify a likely source of their infection. For these reasons, it is becoming increasingly important to be able to identify potential common exposures through DNA fingerprinting of patient isolates.

For foodborne outbreak surveillance to be effective, isolates must be subtyped routinely and the data analyzed promptly at the local level. Clusters can often be detected locally that could not have been identified by traditional epidemiologic methods alone. This is especially true of infections with common pathogens such as *S.* Typhimurium, which occur so frequently that clusters may be hidden among sporadic cases. For *S.* Typhimurium isolates received by the microbiology laboratory at the Minnesota Department of Health from August 14 to September 14, 1995, temporal distribution did not suggest any obvious clustering, but the distribution of PFGE subtypes suggested multiple common sources with continuing exposures (Figure 2). Epidemiologic investigation ultimately linked three of the subtypes to local restaurants, where exposure to *S.* Typhimurium occurred throughout the month (Jeffrey B. Bender, pers. comm.). Without subtyping data, it would have been very difficult to associate cases with exposures occurring over such a prolonged period.

In September 1998, the Minnesota state public health laboratory informed other PulseNet laboratories that it was investigating two clusters of *Shigella sonnei* infections associated with restaurants in Minnesota and asked if other states had observed increases in *S. sonnei* infections or *S. sonnei* isolates with the outbreak PFGE pattern. The Los Angeles County public health department immediately responded that it was also investigating restaurant-associated outbreaks of *S. sonnei* and that the PFGE pattern of their outbreak strain was very similar to the Minnesota pattern. Epidemiologic and laboratory investigations ultimately determined that outbreaks in Massachusetts, Florida, and two Canadian provinces were linked to the Minnesota and Los Angeles outbreaks. With the assistance of the FDA's outbreak trace-back and coordination group, parsley imported from Mexico was identified as the common vehicle (22). Mexican and U.S. authorities inspected the parsley farm and recommended changes in growing and harvesting practices to prevent recurrence of the problem. Rapid sharing of PFGE subtyping data through PulseNet played a critical role in linking these apparently unrelated outbreaks and identifying a common vehicle.

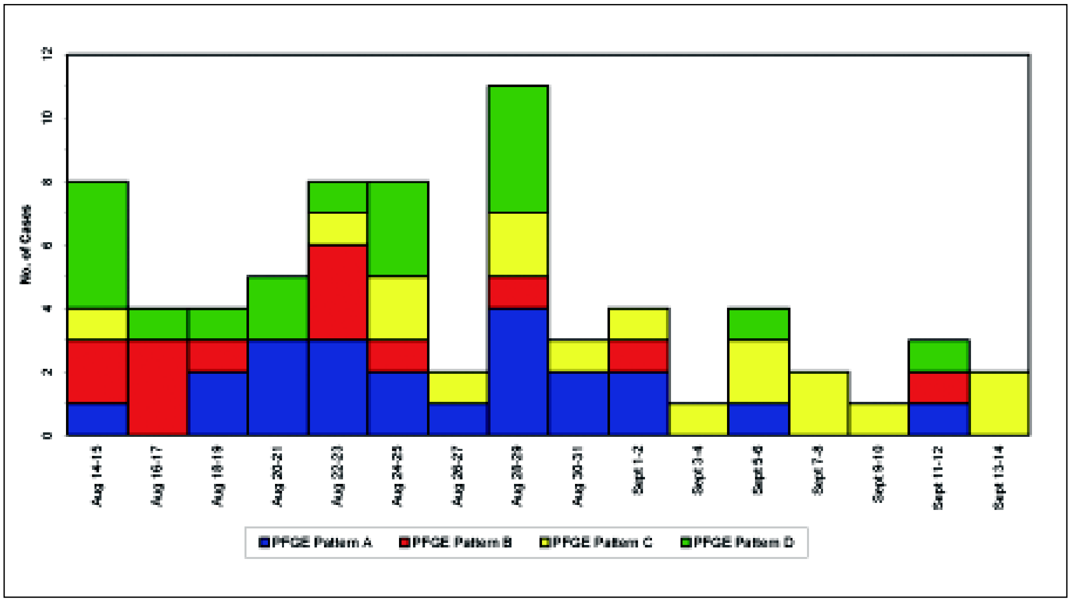

Figure 2. Distribution by date and pulsed-field gel electrophoresis subtype of *Salmonella* ser. Typhimurium isolates received by the Minnesota Department of Health, August 14-September 14, 1995. Data provided by Jeffrey Bender and John Besser, Minnesota Department of Health.

The use of molecular subtyping as part of routine surveillance has benefits beyond outbreak detection. Temporal clustering of unrelated cases is not uncommon, and without molecular subtyping, valuable public health resources can be wasted investigating pseudo-outbreaks. In June and July 1994, an outbreak of *E. coli* O157:H7 infections was suspected when the New Jersey Department of Health and Senior Services received reports of 48 culture-confirmed cases; only four were reported during the same period in 1993 (23). PFGE subtyping found most isolates to have unique patterns, indicating that a large outbreak was unlikely. The probable reason for the sudden increase in case reports was the concomitant increase in the number of laboratories culturing stools for *E. coli* O157:H7 (Figure 3).

Although PulseNet has proven invaluable in detecting foodborne disease outbreaks and facilitating their investigation, molecular subtyping is an adjunct to epidemiologic investigation and not a replacement for it. The observation that isolates from two or more persons have indistinguishable PFGE patterns should not be considered proof that the persons had a common exposure, merely that the isolates in question share a common ancestry. Moreover, outbreaks can be caused by more than one subtype, so that differences in PFGE pattern alone cannot prove that isolates did not have a common source (24,25).

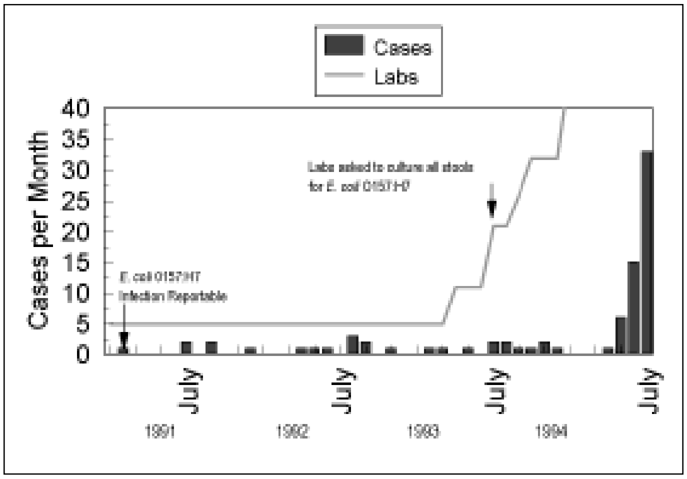

Figure 3. Reported cases of culture-confirmed *Escherichia coli* O157:H7 infection and percentage of surveyed laboratories routinely testing all stool specimens for *E. coli* O157:H7, New Jersey, January 1991 through July 1994 (10).

### Requirements for Effective Functioning

Although the area laboratories are set up to assist neighboring state public health laboratories that are not PulseNet participants, every state must have PFGE subtyping capacity for optimum performance of the network. A dramatic indication of this was provided during the 1997 ground beef-associated *E. coli* O157:H7 outbreak in Colorado. When the outbreak pattern was posted on PulseNet ListServ, most laboratories that were network participants responded within 48 hours that they had no matching PFGE patterns from recent *E. coli* O157:H7 isolates. In contrast, it took more than 2 months to identify a case in Kentucky (not a PulseNet participant state in 1997) that was related to the Colorado outbreak. The Association of Public Health Laboratories has determined that PulseNet participation is a core capacity for all state and territorial public health departments in the United States.

For the network to work efficiently in detecting foodborne disease outbreaks through routine surveillance, PulseNet laboratories must perform, at a minimum, routine PFGE subtyping of *E. coli* O157:H7 and *L. monocytogenes* as soon as isolates are received. In addition, they must perform PFGE subtyping of other foodborne pathogenic bacteria (*Campylobacter jejuni* and *C. coli*, *Salmonella* serotypes, *Shigella* spp., *Bacillus cereus*, *Vibrio cholerae*, *V. parahaemolyticus*, *V. vulnificus*, *Clostridium botulinum*, *C. perfringens*, *Yersinia enterocolitica*) rapidly when the number of isolates received by the laboratory exceeds the expected number for that period. Unfortunately, microbiologists at state and local public health laboratories often have responsibilities for all pathogenic bacteria and may not be able to type incoming isolates of foodborne pathogenic bacteria in a timely manner. In addition, like public health surveillance in general, PulseNet depends on physicians requesting culture of patient specimens if a bacterial infection is suspected and the clinical diagnostic laboratory promptly forwarding isolates to the public health laboratory for typing.

PulseNet relies on the cooperation of all participants in typing foodborne pathogenic bacteria by strict adherence to the standard protocol. Without such a total commitment, PFGE patterns from different PulseNet laboratories could not be compared to ascertain which cases are associated with a specific outbreak. The importance of this was underscored by a recent experience. One PulseNet laboratory had decided to change the PulseNet protocol for *S.* Typhimurium and was using a variation of the standard protocol. A cluster of *S.* Typhimurium cases was detected in that state, and PFGE analysis confirmed that many of the isolates were indistinguishable. However, when attempts were made to determine if an increase in *S.* Typhimurium infections in neighboring states were related to the cluster, the PFGE patterns could not be compared. This caused a delay of several days in the investigation because isolates from the first state had to be re-typed by the standardized protocol.

### Cost-Benefit Analysis

Elbasha et al. recently compared the costs and benefits of PulseNet's molecular subtyping-based surveillance system, using as an example the Colorado state public health laboratory's investigation of the 1997 *E. coli* O157:H7 outbreak in which contaminated frozen hamburger patties were implicated (26). If 15 cases were averted by the recall of potentially contaminated ground beef, the PulseNet system in Colorado would have recovered all costs of start-up and 5 years of operation. These authors point out that the system becomes even more cost-effective if one takes into account resources that would have been wasted to investigate apparent increases in sporadic cases of *E. coli* O157:H7 infections.

### The Growth and Future of PulseNet

Within a very short time, PulseNet has grown beyond expectations and has convincingly demonstrated its effectiveness as a tool for foodborne disease surveillance. It began with one pathogen (*E. coli* O157:H7) and 10 participating laboratories in 1996 that submitted 191 PFGE patterns to the PulseNet database during that year. In 1999, four pathogens (*E. coli* O157:H7, *Salmonella*, *Shigella*, and *Listeria monocytogenes*) were tracked through PulseNet and >9,500 patterns were submitted to the PulseNet database. State and local public health laboratories contributed >78% of the patterns to the PulseNet database (Figure 4). As the FDA are increasing the number of laboratories that perform PFGE subtyping using PulseNet protocols, and the USDA are setting up their own PulseNet-compatible local networks, their contributions to the PulseNet database will no doubt substantially increase. In addition, the representation of PFGE patterns in the PulseNet databases will increase for pathogenic bacteria isolated from foods.

As more public health laboratories at the local and state levels join PulseNet, the role of the area laboratories is changing. The area laboratories provide training and consultation to neighboring PulseNet laboratories, coordinate multistate outbreak investigations when requested, and provide surge capacity for neighboring PulseNet laboratories. Three additional area laboratories, in Michigan, Utah, and Virginia, were designated in 2000, bringing the total number to seven.

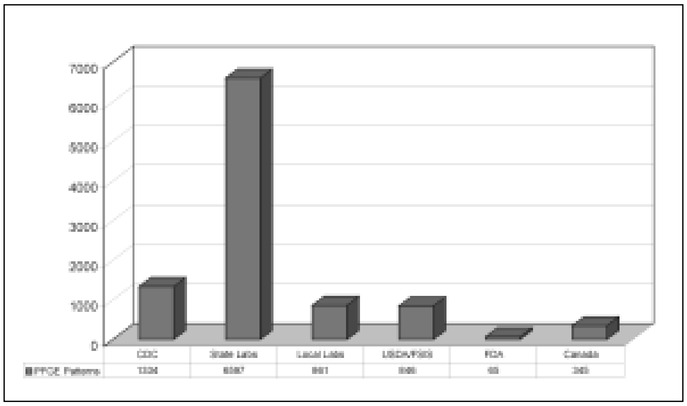

Figure 4. Sources of pulsed-field gel electrophoresis patterns submitted to PulseNet in 1999.

Canada is already an active participant, and international expansion of the network with partners in Mexico, South America, and Europe is anticipated. The long-term vision for PulseNet is a global network of public health laboratories working with food regulatory agencies and industry to improve food safety worldwide.

Finally, we recognize that the methods currently used for subtyping and data analysis will not always be state-of-the-art. We are working to develop, evaluate, and validate DNA sequencing-based subtyping methods for foodborne pathogens. These methods will be gradually implemented in the network, and compatibility will be maintained with existing PFGE data. We are also working with software developers to implement new versions of pattern analysis software and DNA sequence comparison software to improve pattern matching, automate pattern normalization and sequence alignment, and reduce subjectivity in subtype comparisons.

### Acknowledgments

We thank Mike Hoekstra for suggestions on data presentation and Susan Van Duyne for information on the quality assurance and control program for PulseNet; we thank personnel in all participating laboratories for their enthusiastic participation in PulseNet and for sharing their data and findings in a timely manner; and Jeffrey Koplan, James Hughes, Joseph McDade, Mitchell Cohen, and Patrick McConnon for their support of PulseNet.

PulseNet is supported by appropriations to CDC under the National Food Safety Initiative and the Emerging Infectious Diseases Program and by appropriations from various states to their respective public health departments.

Dr. Swaminathan is chief of the Foodborne and Diarrheal Diseases Laboratory Section, CDC, and the principal architect of PulseNet.

### References

1. Holmberg SD, Wachsmuth IK, Hickman-Brenner FW, Cohen ML. Comparison of plasmid profile analysis, phage typing, and antimicrobial susceptibility testing in characterizing *Salmonella typhimurium* isolates from outbreaks. J Clin Microbiol 1984;19:100-4.

2. Holmberg SD, Wachsmuth K. Plasmid and chromosomal DNA analyses in the epidemiology of bacterial diseases. In: Swaminathan B, Prakash G, editors. Nucleic acid and monoclonal antibody probes: applications in diagnostic microbiology. New York: Marcel Dekker; 1989. p. 105-29.
3. Ackers ML, Mahon BE, Leahy E, Goode B, Damrow T, Hayes PS, et al. An outbreak of *Escherichia coli* O157:H7 infections associated with leaf lettuce consumption. J Infect Dis 1998;177:1588-93.
4. Barrett TJ. Molecular fingerprinting of foodborne pathogenic bacteria: An introduction to methods, uses and problems. In: Tortorello ML, Gendel SM, editors. Food microbiological analysis: new technologies. New York: Marcel Dekker; 1997. p. 249-64.
5. Graves LM, Swaminathan B, Hunter SB. Subtyping *Listeria monocytogenes*. In: Ryser EM, Marth EH, editors. *Listeria*, listeriosis and food safety. New York: Marcel Dekker; 1999. p. 279-98.
6. Jimenez A, Barros-Velazquez J, Rodriguez J, Villa TG. Restriction endonuclease analysis, DNA relatedness and phenotypic characterization of *Campylobacter jejuni* and *Campylobacter coli* isolates invovled in food-borne disease. J Appl Microbiol 1997;82:713-21.
7. Maslanka SE, Kerr JG, Williams G, Barbaree JM, Carson LA, Miller JM, et al. Molecular subtyping of *Clostridium perfringens* by pulsed-field gel electrophoresis to facilitate food-borne-disease outbreak investigations. J Clin Microbiol 1999;37:2209-14.
8. Threlfall EJ, Hampton MD, Ward LR, Rowe B. Application of pulsed-field gel electrophoresis to an international outbreak of *Salmonella agona*. Emerg Infect Dis 1996;2:130-2.
9. Threlfall EJ, Ward LR, Hampton MD, Ridley AM, Rowe B, Roberts D, et al. Molecular fingerprinting defines a strain of *Salmonella enterica* serotype Anatum responsible for an international outbreak associated with formula-dried milk. Epidemiol Infect 1998;121:289-93.
10. Wachsmuth K. Molecular epidemiology of bacteria infections: Examples of methodology and of investigations of outbreaks. Rev Infect Dis 1986;8:682-92.
11. Swaminathan B, Matar GM. Molecular typing methods. In: Persing DH, Smith TF, Tenover FC, White TJ, editors. Diagnostic molecular microbiology. Washington: American Society for Microbiology; 1993. p. 26-50.
12. Barrett TJ, Lior H, Green JH, Khakhria R, Wells JG, Bell BP, et al. Laboratory investigation of a multi-state food-borne outbreak of *Escherichia coli* O157:H7 by using pulsed-field gel electrophoresis and phage typing. J Clin Microbiol 1994;32:3013-7.
13. Stephenson J. New approaches for detecting and curtailing foodborne microbial infections. JAMA 1997;277:1337-40.
14. Centers for Disease Control and Prevention. Addressing emerging infectious disease threats: a prevention strategy for the United States. Atlanta: U.S. Department of Health and Human Services; 1994.
15. Angulo FJ, Voetsch AC, Vugia D, Hadler JL, Farley M, Hedberg C, et al. Determining the burden of human illness from food borne diseases. CDC's emerging infectious disease program Food Borne Diseases Active Surveillance Network (FoodNet). Vet Clin North Am Food Anim Pract 1998;14:165-72.
16. Gautom RK. Rapid pulsed-field gel electrophoresis protocol for *E. coli* O157:H7 and other Gram-negative organisms in one day. J Clin Microbiol 1997;35:2977-80.
17. Centers for Disease Control and Prevention. Standardized molecular subtyping of foodborne bacterial pathogens by pulsed-field gel electrophoresis: a manual. Atlanta: National Center for Infectious Diseases; 1996 (updated 2000).
18. Cody SH, Glynn MK, Farrar JA, Cairns KL, Griffin PM, Kobayashi J, et al. An outbreak of *Escherichia coli* O157:H7 infection from unpasteurized commercial apple juice. Ann Intern Med 1999;130:202-9.
19. Centers for Disease Control and Prevention. Multistate outbreak of *Salmonella* serotype Agona infections linked to toasted oats cereal—United States, April-May, 1998. MMWR Morb Mortal Wkly Rep 1998;47:462-4.
20. Centers for Disease Control and Prevention. Multistate outbreak of listeriosis—United States, 1998. MMWR Morb Mortal Wkly Rep 1998;47:1085-6.
21. Centers for Disease Control and Prevention. Update: Multistate outbreak of listeriosis—United States, 1998-1999. MMWR Morb Mortal Wkly Rep 1999;47:1117-8.
22. Centers for Disease Control and Prevention. Outbreaks of *Shigella sonnei* infection associated with eating fresh parsley—United States and Canada, July-August, 1998. MMWR Morb Mortal Wkly Rep 1999;48:285-9.
23. Mead P, Finelli L, Lambert-Fair MA, Champ D, Townes J, Hutwagner L, et al. Risk factors for sporadic infection with *Escherichia coli* O157:H7. Arch Intern Med 1997;157:204-8.
24. Keene W, Hedberg K, Herriott DE, Hancock DD, McKay RW, Barrett TJ, et al. A prolonged outbreak of *Escherichia coli* O157:H7 infections caused by commercially distributed milk. J Infect Dis 1997;176:815-8.
25. Jackson LA, Keene WE, McAnulty JM, Alexander ER, Diermayer M, Davis MA, et al. Where's the beef? The role of cross-contamination in four chain restaurant-associated outbreaks of *Escherichia coli* O157:H7 in the Pacific Northwest. Ann Intern Med 2000;160:2380-5.
26. Elbasha EH, Fitzsimmons TD, Meltzer MI. Costs and benefits of a subtype-specific surveillance system for identifying *Escherichia coli* O157:H7 outbreaks. Emerg Infect Dis 200;6:293-7.

## References

[PDF_00727] Ackers M. L., Mahon B. E., Leahy E., Goode B., Damrow T., Hayes P. S., Bibb W. F., Rice D. H., Barrett T. J., Hutwagner L. (1998). An outbreak of Escherichia coli O157:H7 infections associated with leaf lettuce consumption.. J Infect Dis.

[PDF_00770] Angulo F. J., Voetsch A. C., Vugia D., Hadler J. L., Farley M., Hedberg C., Cieslak P., Morse D., Dwyer D., Swerdlow D. L. (1998). Determining the burden of human illness from food borne diseases. CDC's emerging infectious disease program Food Borne Diseases Active Surveillance Network (FoodNet).. Vet Clin North Am Food Anim Pract.

[PDF_00761] Barrett T. J., Lior H., Green J. H., Khakhria R., Wells J. G., Bell B. P., Greene K. D., Lewis J., Griffin P. M. (1994). Laboratory investigation of a multistate food-borne outbreak of Escherichia coli O157:H7 by using pulsed-field gel electrophoresis and phage typing.. J Clin Microbiol.

[PDF_00786] Centers for Disease Control and Prevention (CDC) (1998). Multistate outbreak of Salmonella serotype Agona infections linked to toasted oats cereal--United States, April-May, 1998.. MMWR Morb Mortal Wkly Rep.

[PDF_00790] Centers for Disease Control and Prevention (CDC) (1998). Multistate outbreak of listeriosis--United States, 1998.. MMWR Morb Mortal Wkly Rep.

[PDF_00796] Centers for Disease Control and Prevention (CDC) (1999). Outbreaks of Shigella sonnei infection associated with eating fresh parsley--United States and Canada, July-August 1998.. MMWR Morb Mortal Wkly Rep.

[PDF_00793] Centers for Disease Control and Prevention (CDC) (1999). Update: multistate outbreak of listeriosis--United States, 1998-1999.. MMWR Morb Mortal Wkly Rep.

[PDF_00782] Cody S. H., Glynn M. K., Farrar J. A., Cairns K. L., Griffin P. M., Kobayashi J., Fyfe M., Hoffman R., King A. S., Lewis J. H. (1999). An outbreak of Escherichia coli O157:H7 infection from unpasteurized commercial apple juice.. Ann Intern Med.

[PDF_00775] Gautom R. K. (1997). Rapid pulsed-field gel electrophoresis protocol for typing of Escherichia coli O157:H7 and other gram-negative organisms in 1 day.. J Clin Microbiol.

[PDF_00715] Holmberg S. D., Wachsmuth I. K., Hickman-Brenner F. W., Cohen M. L. (1984). Comparison of plasmid profile analysis, phage typing, and antimicrobial susceptibility testing in characterizing Salmonella typhimurium isolates from outbreaks.. J Clin Microbiol.

[PDF_00807] Jackson L. A., Keene W. E., McAnulty J. M., Alexander E. R., Diermayer M., Davis M. A., Hedberg K., Boase J., Barrett T. J., Samadpour M. (2000). Where's the beef? The role of cross-contamination in 4 chain restaurant-associated outbreaks of Escherichia coli O157:H7 in the Pacific Northwest.. Arch Intern Med.

[PDF_00737] Jiménez A., Barros-Velázquez J., Rodríguez J., Villa T. G. (1997). Restriction endonuclease analysis, DNA relatedness and phenotypic characterization of Campylobacter jejuni and Camp. coli isolates involved in food-borne disease.. J Appl Microbiol.

[PDF_00803] Keene W. E., Hedberg K., Herriott D. E., Hancock D. D., McKay R. W., Barrett T. J., Fleming D. W. (1997). A prolonged outbreak of Escherichia coli O157:H7 infections caused by commercially distributed raw milk.. J Infect Dis.

[PDF_00742] Maslanka S. E., Kerr J. G., Williams G., Barbaree J. M., Carson L. A., Miller J. M., Swaminathan B. (1999). Molecular subtyping of Clostridium perfringens by pulsed-field gel electrophoresis to facilitate food-borne-disease outbreak investigations.. J Clin Microbiol.

[PDF_00800] Mead P. S., Finelli L., Lambert-Fair M. A., Champ D., Townes J., Hutwagner L., Barrett T., Spitalny K., Mintz E. (1997). Risk factors for sporadic infection with Escherichia coli O157:H7.. Arch Intern Med.

[PDF_00765] Stephenson J. (1997). New approaches for detecting and curtailing foodborne microbial infections.. JAMA.

[PDF_00746] Threlfall E. J., Hampton M. D., Ward L. R., Rowe B. (1996). Application of pulsed-field gel electrophoresis to an international outbreak of Salmonella agona.. Emerg Infect Dis.

[PDF_00749] Threlfall E. J., Ward L. R., Hampton M. D., Ridley A. M., Rowe B., Roberts D., Gilbert R. J., Van Someren P., Wall P. G., Grimont P. (1998). Molecular fingerprinting defines a strain of Salmonella enterica serotype Anatum responsible for an international outbreak associated with formula-dried milk.. Epidemiol Infect.

[PDF_00754] Wachsmuth K. (1986). Molecular epidemiology of bacterial infections: examples of methodology and of investigations of outbreaks.. Rev Infect Dis.

